# The development and validation of a family functioning measure for Aboriginal and Torres Strait Islander adults

**DOI:** 10.1186/s12889-022-14363-7

**Published:** 2022-10-28

**Authors:** Makayla-May Brinckley, Roxanne Jones, Philip J. Batterham, Alison L. Calear, Raymond Lovett

**Affiliations:** 1 National Centre for Epidemiology and Population Health, Research School of Population Health, Australian National University, 54 Mills Road, Acton, 2601 Australia; 2 Wiradjuri, Australia; 3 Palawa, Australia; 4 Centre for Mental Health Research, Research School of Population Health, Australian National University, 63 Eggleston Road, Acton, 2601 Australia; 5 Ngiyampaa (Wongaibon), Australia

**Keywords:** Psychometrics, Reliability, Validity, Measurement, Tool development, Family wellbeing, Family functioning, Aboriginal and Torres Strait Islander, Indigenous

## Abstract

**Background:**

Family and kinship networks are a key aspect of culture for Aboriginal and Torres Strait Islander peoples from Australia. They are intrinsically connected to good health and wellbeing, and cultural knowledge exchange. However, despite the known importance of family and kinship networks in Aboriginal and Torres Strait Islander cultures, and the move towards family-centred approaches in healthcare service provision, there is no validated, national measure of family functioning for Aboriginal and Torres Strait Islander peoples. A valid tool to measure family functioning is necessary in order to better understand what fosters good family functioning, and to inform and develop programs and healthcare interventions.

**Methods:**

Mayi Kuwayu: the National Study of Aboriginal and Torres Strait Islander Wellbeing is a longitudinal cohort study of Aboriginal and Torres Strait Islander adults aged 16 years and over. An existing family functioning scale was modified for use in the Mayi Kuwayu Study to measure family functioning at the national Aboriginal and Torres Strait Islander population level. This study used a national sample of Aboriginal and Torres Strait Islander adults (*N =* 8705, ≥16 years) for the psychometric assessment of the modified Mayi Kuwayu Study Family Functioning Measure. This involved face validity, acceptability, internal consistency/reliability, construct validity, and convergent and divergent validity testing.

**Results:**

Participants in this study were 8705 Aboriginal and Torres Strait Islander peoples, with a mean age of 48 years, who primarily live in regional Australia (47.3%). The Mayi Kuwayu Family Functioning Measure demonstrated face validity for family functioning and had good internal consistency/reliability (Cronbach’s alpha > 0.90). Construct validity results were mixed, with an indication of uni-dimensionality (with one component explaining 59.5% of variance), but some item redundancy and inconsistency in responding patterns among groups of respondents. Balancing psychometric properties with Aboriginal and Torres Strait Islander expert and end-user feedback of the measure indicate that the full scale should be retained. Finally, the measure demonstrated strong convergent and divergent validity, with prevalence ratios exhibiting dose-response relationships between family functioning and conceptually related outcomes (convergent validity) and conceptually unrelated outcomes (divergent validity).

**Conclusion:**

The Mayi Kuwayu Family Functioning Measure is a valid measure of family functioning in the Aboriginal and Torres Strait Islander adult population.

**Supplementary Information:**

The online version contains supplementary material available at 10.1186/s12889-022-14363-7.

## Background

For Aboriginal and Torres Strait Islander peoples, family and kinship networks are a key aspect of culture. Family is a “fluid and complex composition based on overlapping kinship systems and networks” [1]. Kinship systems are a network of social relationships that includes the immediate and extended family and can also include other community members, based on responsibilities of duty and care [2]. Strong family units help children form social networks, provide children with resources and care, and teach children about the world around them [3]. Family and kinship systems are based on both biological and social networks, and are important in cultural transfer and the overall health and wellbeing of Aboriginal and Torres Strait Islander peoples. Previous work demonstrates that strong family wellbeing is a crucial aspect of good wellbeing overall for Aboriginal and Torres Strait Islander families. For example, a study of Aboriginal children in Melbourne, Australia, found that being closely connected to family, kinship and community is critical for staying connected to culture and maintaining good wellbeing outcomes. Participants in this study stressed that health is important for the overall wellbeing of their children, but their connection to family and community and their role in passing on cultural knowledge is equally – if not more – important [4].

Healthcare providers are increasingly using family-centred care in supporting and caring for the health of their Indigenous clients internationally [5]. This approach sees individuals as embedded in their broader family unit and provides services around all individuals (adults and children) within the family, rather than just one individual [5]. In a review of 18 studies, family-centred interventions were found to improve Aboriginal and Torres Strait Islander children’s health, and the health and parenting knowledge of their caregivers. This evidence is relatively new, so a limited number of studies evaluated the effectiveness of interventions; however, it does indicate that family wellbeing is linked to improved health and wellbeing outcomes [5]. This emerging evidence shows us the importance of family functioning and wellbeing for health and social factors, and that more and more healthcare services are recognising the importance of healthcare for not just the individual, but also their whole family.

Despite this known importance of Aboriginal and Torres Strait Islander family functioning and wellbeing, there is currently no validated, national measure of family functioning for Aboriginal and Torres Strait Islander peoples in Australia [3]. One known measure of family functioning is the Western Australian Aboriginal Child Health Survey (WAACHS). The WAACHS developed a family functioning scale for use in Aboriginal communities in Western Australia. The 9-item scale was designed to measure “the extent to which families have established a climate of co-operation, emotional support and good communication” [6]. The scale was created using family and resilience protective factors identified through a literature review of international research on family resilience [7]. The wording used in the WAACHS family functioning scale was developed in collaboration with Aboriginal health professionals to ensure it was able to be interpreted by respondents whose first language was Aboriginal English or an Aboriginal language [8].

WAACHS family functioning scale items are scored and summed, with total scores split into quartiles: “Poor” (score = 9-34), “Fair” (score = 35-38), “Good” (score = 39-41) and “Very Good” (score = 42-45). The authors acknowledge that these quartiles are labelled “somewhat arbitrarily” [6]. Limited psychometric testing has been conducted on the WAACHS family functioning scale. A factor analysis of the scale indicated that it has a unitary factor structure, and a Euclidean distance model was fitted to determine the closeness of items, which again confirmed the one factor structure [6]. Significant associations were found between poor family functioning and financial strain, alcohol use, not having someone to yarn to (converse respectfully in a safe space), and carer relationship issues. There were no significant associations found between family functioning and asthma, hearing problems, mobility issues, or other physical health problems [8].

The WAACHS family functioning scale is the only known Aboriginal family functioning measure. The scale was developed based on identified family and resilience protective factors from literature review, but it was not developed for use in a national context. We do not currently have a valid measure to assess Aboriginal and Torres Strait Islander family functioning, or for monitoring Aboriginal and Torres Strait Islander family wellbeing programs nationally. Family is a key cultural domain for Aboriginal and Torres Strait Islander peoples and therefore it is important for Aboriginal and Torres Strait Islander peoples to monitor family functioning, and to identify factors linked to increased family functioning [1].

The aim of this paper is to provide a psychometric assessment of a modified measure of family functioning for the national Aboriginal and Torres Strait Islander population, using data from Mayi Kuwayu: the National Study of Aboriginal and Torres Strait Islander Wellbeing (the Mayi Kuwayu Study). Acceptability, internal consistency/reliability, construct validity, and convergent and divergent validity will be assessed. This paper is not intended to describe the prevalence of family functioning in the cohort overall or by demographic (or other) factors, nor is it intended to provide evidence on associations between family functioning and health or other outcomes. This will be the focus of an additional paper once psychometric properties of the scale have been established.

## Method

### Study population

The Mayi Kuwayu Study is a national longitudinal cohort study of Aboriginal and Torres Strait Islander adults aged 16 years and over. Participants are recruited through a multi-mode approach, via a mail-out survey, through in-community recruitment (including on-the-ground community researchers), community partnerships, online recruitment, over-the-phone, or through word of mouth [9]. Questionnaires are self-completed on paper or online, or completed with assistance from community researchers or study partners. Data used in this validation study are from the baseline rolling data collection (Data Release 3.0, *N =* 9843) whose survey data was processed between October 2018 to December 2020. Responses are restricted to Mayi Kuwayu Study participants with a total family functioning score (*N =* 8705). All data in this study are based on self-reported responses to the questionnaire. Details of the study design are provided elsewhere [9].

### Aboriginal and Torres Strait Islander governance

The Mayi Kuwayu Study, and the present validation study, are governed by the *Thiitu Tharrmay Aboriginal and Torres Strait Islander Governance Committee*. While it is not possible to represent the full diversity of the Aboriginal and Torres Strait Islander population, members of Thiitu Tharrmay collectively represent a diversity of Aboriginal and Torres Strait Islander lived experiences, come from different communities, cultures and Countries, and different research backgrounds and expertise. Thiitu Tharrmay consists of at least 10 Aboriginal and/or Torres Strait Islander members who are involved in the analyses, interpretations and outputs of work conducted by the Mayi Kuwayu Study, including the present study.

### Development of the Mayi Kuwayu study family functioning measure

The Mayi Kuwayu Study modified the WAACHS family functioning scale for use in its questionnaire, as it was the only known family functioning scale for Aboriginal and Torres Strait Islander peoples available. Modification and extensive face validity testing occurred though 28 focus groups with 197 Aboriginal and Torres Strait Islander peoples [9, 10]. Participants were aged from 16 years to over 70 years old, and represented saltwater, freshwater, desert and Island Aboriginal and Torres Strait Islander mobs across urban, regional and remote Australia [10]. Ensuring diverse voices were captured in this process was essential, as the WAACHS was developed only for use in Western Australian Aboriginal communities, while the Mayi Kuwayu Study family functioning measure was being modified for widespread use at the Aboriginal and Torres Strait Islander population level. See Supplementary file 1 (Table S1) for full focus group participant details.

Focus groups were conducted through an iterative process, where wording was developed by Aboriginal and Torres Strait Islander participants in focus groups, re-tested in subsequent focus groups, and revised if needed. The language of the measure was adapted to reduce wordiness, increase Aboriginal and Torres Strait Islander participant understanding and cultural relevance, while maintaining the underlying family and resilience protective concepts as those in the WAACHS scale [7]. No concepts relevant to family functioning additional to those already covered in WAACHS existing items were identified by participants. Testing and re-testing of the language of the measure ended at data saturation; that is, when no new information was produced.

Through this face validity assessment, the WAACHS scale was modified to the Mayi Kuwayu Study Family Functioning Measure (FFM) (see Table 1 for item comparison).

**Table 1:** Modification of the WAACHS family functioning scale for the development of the Mayi Kuwayu Family Functioning Measure (FFM)

WAACHS family functioning scale^**a**^	Mayi Kuwayu Study FFM
Stem: *Here are some statements about families. How well do these match the way things are done in your family?*	Stem: *In my family …*
The way we get on together helps us to cope with the hard times	We get on together and cope in the hard times
We like to remember people’s birthdays and celebrate other special events	We celebrate special days/events
We find it easy to talk with each other about the things that really matter	We talk with each other about the things that matter
We are always there for each other and know that the family will survive no matter what	We are always there for each other
When it comes to managing money we are careful and make good decisions	We manage money well
Our family has a lot in common in the interests we share and the things we do	We have common interests
People in our family are accepted for who they are	People are accepted for who they are
We have good support from our in-laws, relatives and friends	We have good support from mob
We have family traditions and customs we would like to pass on to our children	We have family knowledge and traditions that we pass on to our children

^a^ (Silburn et al., 2006)

### Measures

The FFM asks participants to rate the extent to which they agree with a set of nine statements (Table 1). Response options are “not at all” (score = 1), “a little bit” (score = 2), “a fair bit” (score = 3), “a lot” (score = 4), or “unsure” (recoded to missing).

For participants who responded unsure or missing to one item only, an imputed value (the mean of that participant’s other eight FFM items) replaced the missing or unsure response. Our aim for imputation was to keep the scoring of the scale as ecologically valid as possible and have the scale validated in the way it could be widely *used* in Aboriginal and Torres Strait Islander communities, rather than adopting an “ideal” approach. Individual mean imputation is considered to be simpler and easier to understand than multiple imputation, and is a “more intuitive approach to imputing values”, while still producing appropriate results [11]. We opted for individual mean imputation of one item only, rather than multiple imputation, as it is not feasible for multiple imputation to be done each time the scale is used in Aboriginal and Torres Strait Islander communities. Therefore, for the purpose of the FFM, individual mean imputation is most appropriate way to maintain the utility (usefulness) of the measure over other forms of imputation.

We test differences in the sample in terms of the outcome for non-imputed and individual mean imputed values to determine whether this method has a significant impact on results. We decided a priori that if a significant difference in the sample across demographic outcomes (age group, gender, remoteness, Indigeneity) was found, we would not use the individual mean imputation method.

A total family functioning score is created by summing responses to the nine items. The total family functioning score is recoded to missing if more than one of the individual items are “missing” or “unsure”. In line with the WAACHS methods, quartiles are utilised, with categories labelled as “Low family functioning” (scores: 9 to ≤24), “Moderate family functioning” (> 24 to ≤29), “High family functioning” (> 29 to ≤33) and “Very high family functioning” (> 33 to 36).

Other variables used for validation were selected a priori based on literature and input from Thiitu Tharrmay. For convergent validity, we used good family financial security as this was found to have a strong association with family functioning measured by the WAACHS [8], and we use experience of pain as identified by authors and Thiitu Tharrmay. Experience of pain is not limited to physical pain, but encompasses all aspects of social and emotional wellbeing, as identified in ongoing internal validation work. Pain was selected as Aboriginal and Torres Strait Islander peoples experience pain in holistic ways that can relate to all aspects of life, including family functioning.

Conceptually, family functioning is potentially related to most variables in the Mayi Kuwayu Study dataset. For divergent validity, we used cardiovascular disease (CVD), as a measure conceptually expected to be less strongly related to family functioning than measures selected for convergent validity. Full details of all measures are described in Supplementary file 1 (Table S2).

### Analysis

#### Participant characteristics

Participants were described by age group (16-24, 25-34, 35-44, 45-54, 55-64, ≥65), gender (men, women, other genders), remoteness (major cities, regional, remote/very remote), and Indigeneity (Aboriginal, Torres Strait Islander, or both Aboriginal and Torres Strait Islander). Distribution of responses to individual family functioning items were described overall and by age group, gender, remoteness, and Indigeneity, with ANOVA analysis and Tukey’s post-hoc test indicating significant differences across demographic characteristics (age group, gender, remoteness, and Indigeneity).

#### Acceptability

Acceptability was assessed through examination of missing data across each item and the entire measure. Missing data of less than 10% was considered desirable [12]. We assessed rates of “unsure” versus “sure” responses (i.e., response options not at all, a little bit, a fair bit, and a lot) across demographics, with total scores summing to 100% for “sure” and 100% for “unsure” responses, in order to understand characteristics of people who did and did not complete the measure to determine if these may have been influenced by selection biases.

### Statistical analyses

The sample was randomly split into two subsamples to enable scale development and validation to be conducted independently [13, 14]. Internal consistency/reliability was assessed using Cronbach’s alpha on both sub-samples, with acceptable scores at alpha ≥0.70 [15].

Construct validity relates to how well scores on the scale are indicative of the underlying construct. We tested this primarily by using factor analysis to evaluate whether the items in the scale formed a single dimension of family functioning. Construct validity was assessed using the split-sample method for development and validation of the scale’s factor structure to first explore the factor structure and then confirm the factors. This method was selected because the FFM is a new measure at the Aboriginal and Torres Strait Islander population level, and because psychometric properties of the WAACHS have not been tested previously [14]. Sample 1 (development) used Exploratory Factor Analysis (EFA) running a Principal Component Analysis (PCA) and Factor Analysis. Sample 2 (validation) used Confirmatory Factor Analysis (CFA), with four fit indices used to assess the fit: root mean square error of approximation (RMSEA), root mean squared residual (SRMR), comparative fit index (CFI) and Tucker-Lewis Index (TLI). A cut-off between .05 and .08 for RMSEA, cut-off less than .08 for SRMR, a cut-off between .90 and .95 for the CFI, and a cut off of .95 for TLI was used as a measure of adequate fit [16, 17]. We then used item response theory to assess whether the response categories were associated with sufficiently distinct scores on the latent construct of family functioning [18].

Convergent validity was tested by quantifying the association of family functioning against theoretically related concepts (family financial security and pain level), and divergent validity was tested by quantifying the association of family functioning against a theoretically unrelated concept (CVD) [19]. We anticipated that as family functioning increases, financial security increases and pain decreases, and that there would be a weak to no relationship between family functioning and CVD. For both convergent and divergent validity, binomial regression was used and for common outcomes prevalence ratios (PR) and 95% Confidence Intervals (CI) were calculated. All analyses were run using STATA 16. An alpha level of 0.05 was considered significant for all analyses.

### Ethics

The Mayi Kuwayu Study is Aboriginal-led, designed, and governed. It is conducted with ethics approval from relevant Aboriginal and Torres Strait Islander organisations and from national, State and Territory Human Research Ethics Committees (HRECs). This study was conducted following operational research policies of the Mayi Kuwayu Study Data Governance Committee (Project D200504), under advice from the Thiitu Tharrmay Aboriginal and Torres Strait Islander reference group, and under the Australian National University HREC protocol 2016/767 (Related File 1).

## Results

### Analysis

#### Participant characteristics

Participants are 8705 Aboriginal and Torres Strait Islander peoples aged 16 years and older. The individual mean imputation method of participants who were missing or unsure on one item only does not significantly change the sample in terms of demographic outcomes (age group, gender, remoteness, Indigeneity) (Supplementary file 1, Table S3). Given that this method does not have a significant impact on results, we report on individual mean imputed results, unless otherwise indicated. Participants are primarily over the age of 45 (58.9%), women (60.2%), and living in regional Australia (47.3%). The mean age of the sample is 48.2 years (SD = 0.18). The majority of participants are Aboriginal (91.3%). The mean FFM score in the total sample is 27.68 (SD = 0.07).

ANOVA analysis indicates significant differences in family functioning scores by age group (*p* < 0.001), gender (*p* = 0.001), and level of remoteness (*p* < 0.001). Tukey’s post hoc analysis indicates significant differences in age groups, with participants aged ≥65 years reporting higher family functioning mean scores than those aged 16-24, 45-54 and 55-64 years. Tukey’s analysis indicates significant differences between women and men, with women reporting higher levels of family functioning than men (mean = 27.92 vs 27.34 respectively; Table 2). Those living in remote or very remote areas of Australia have significantly higher levels of family functioning (mean = 29.55) than those living in major cities (mean = 27.23) or regional areas (mean = 27.66). Finally, ANOVA analysis indicates that family functioning scores across Indigeneity are approaching significance (*p* = 0.048), however Tukey’s post hoc analysis indicates no significant differences between groups (Table 2).

**Table 2:** Distribution of participants by demographic characteristics and assessment of family functioning scores (*N =* 8705)

	n	%	Mean score (95%CI)
**Total FFM score**			27.68 (27.55, 27.82)
**Age group (years)**			
16-24	881	10.1	27.48 (27.03, 27.93)
25-34	1160	13.3	27.85 (27.48, 28.23)
35-44	1283	14.7	27.74 (27.39, 28.09)
45-54	1649	18.9	27.19 (26.86, 27.52)
55-64	2000	23.0	27.50 (27.21, 27.79)
≥ 65	1481	17.0	28.45 (28.12, 28.79)
Missing	251	2.9	–
**Gender**			
Man	3274	37.6	27.34 (27.11, 27.57)
Woman	5243	60.2	27.92 (27.74, 28.10)
Other genders	7	0.1	23.46 (19.63, 27.30)
Missing	181	2.1	–
**Level of remoteness**			
Major city	3594	41.3	27.23 (27.01, 27.45)
Regional	4119	47.3	27.66 (27.45, 27.86)
Remote and very remote	959	11.0	29.55 (29.15, 29.94)
Missing	33	0.4	–
**Indigeneity**			
Aboriginal	7946	91.3	27.64 (27.49, 27.78)
Torres Strait Islander	270	3.1	28.58 (27.75, 29.40)
Aboriginal and Torres Strait Islander	355	4.1	27.99 (27.29, 28.69)
Missing	134	1.5	–

*Range for mean score for total family functioning score is 9-36, where higher scores indicate higher levels of family functioning

#### Acceptability

A total of 9843 Aboriginal and Torres Strait Islander participants were eligible for this study. Before individual mean imputation, 2403 participants (24.4%) were missing or unsure on at least one of the 9 items in the FFM. Each item before imputation had less than 5% missing (Table 3).

**Table 3:** Distribution of responses to family functioning items without individual mean imputation (*N =* 9843)

Family functioning item	Mean score (95%CI)^**a**^	Not at all	A little bit	A fair bit	A lot	Unsure	Missing
		n(%)					
We get on together and cope in the hard times	3.24 (3.22, 3.26)	610 (6.2)	1474 (15.0)	2391 (24.3)	4888 (49.7)	182 (1.9)	298 (3.0)
We celebrate special days/events	3.22 (3.20, 3.24)	688 (7.0)	1600 (16.3)	2282 (23.2)	4847 (49.2)	140 (1.4)	286 (2.9)
We talk with each other about the things that matter	3.16 (3.14, 3.18)	679 (6.9)	1746 (17.7)	2586 (26.3)	4422 (44.9)	133 (1.4)	277 (2.8)
We are always there for each other	3.56 (3.34, 3.38)	514 (5.2)	1237 (12.6)	2120 (21.5)	5560 (56.5)	126 (1.3)	286 (2.9)
We manage money well	2.90 (2.88, 2.92)	855 (8.7)	2137 (21.7)	3289 (33.4)	2977 (30.2)	295 (3.0)	290 (3.0)
We have common interests	3.02 (3.00, 3.04)	738 (7.5)	1898 (19.3)	3122 (31.7)	3479 (35.3)	261 (2.7)	345 (3.5)
People are accepted for who they are	3.37 (3.35, 3.39)	440 (4.5)	1099 (11.2)	2374 (24.1)	5403 (54.9)	228 (2.3)	299 (3.0)
We have good support from mob	2.77 (2.74, 2.80)	1685 (17.1)	1588 (16.1)	1891 (19.2)	3030 (30.8)	1249 (12.7)	400 (4.1)
We have family knowledge and traditions that we pass on to our children	2.62 (2.59, 2.64)	1824 (18.5)	2474 (25.1)	1701 (17.3)	2730 (27.7)	759 (7.7)	355 (3.6)

^**a**^Range for mean score for individual items is 1-4, where higher scores indicate higher levels of family functioning

All items have significant variation in “unsure” versus “sure” responses across remoteness level. Significant differences in “unsure” versus “sure” responses are also found across gender across all items except “We have good support from mob” and “We have family knowledge and traditions that we pass on to our children”. Significant variation across age in “unsure” versus “sure” responses are only found in items “We have common interests”, “We have good support from mob” and “We have family knowledge and traditions that we pass on to our children” (Supplementary file 1, Table S4).

Participants more commonly answered the FFM items with response options “a fair bit” and “a lot”. Items “We are always there for each other” and “People are accepted for who they are” have the highest proportion of “a lot” responses of all the scale items. Items “We have good support from mob” and “We have family knowledge and traditions that we pass on to our children” have the highest proportion of “not at all” and “unsure” responses of all the scale items (Table 3).

After imputation for participants missing or unsure on one item only, 1138 participants (11.6%) are excluded from this study. All results following report individual mean imputed results.

### Statistical analyses

#### Internal consistency/reliability

Cronbach’s α for sub-sample 1 is 0.905 and Cronbach’s α for sub-sample 2 is 0.906.

#### Construct validity

**Fig. 1:**
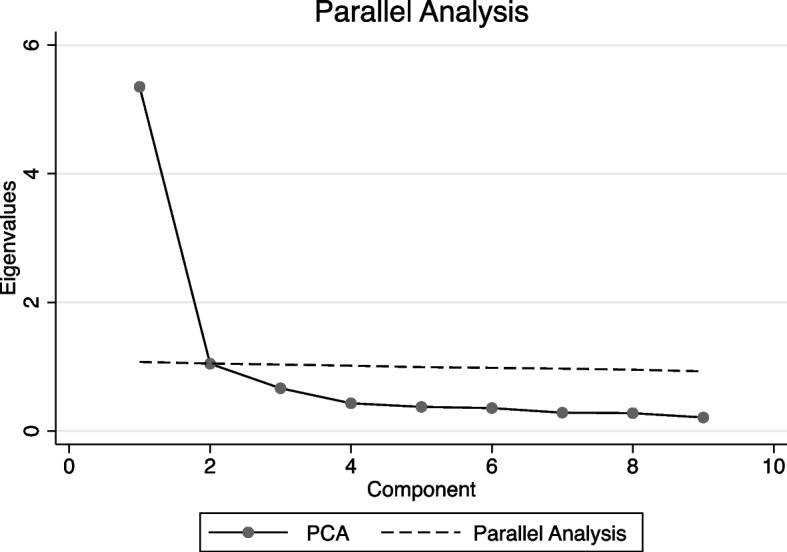
PCA results

EFA is conducted on sub-sample 1 and CFA conducted on sub-sample 2. EFA indicates one component. The PCA also indicates a unidimensional construct (Fig. 1), with 59.5% of the variance explained by one component.

CFA indicates mixed results for the fit of the model: RMSEA = 0.126 is above the recommended threshold, TLI = 0.899 is just below recommended threshold of > 0.95, and SRMR = 0.060 and CFI = 0.924 within the guidelines for adequate fit. As the RMSEA is a measure of fit of the model, our high RMSEA result indicates that the model is not a good fit. Modification indices indicate model improvement if two pairs of error terms are correlated (“support from mob” and “knowledge”). The model was rerun with the error terms correlated. The fit of the model was improved, with SRMR = 0.026, CFI = 0.976, TLI = 0.966 all within guidelines for good fit, while RMSEA = 0.073 indicating adequate fit. Overall, there is good indication of uni-dimensionality, although evidence of some item redundancy (items “support from mob” and “knowledge”) in the scale based on local dependence. Item discriminability is the ability of an item to differentiate among individuals on the basis of the underlying construct of family functioning. All items have good discrimination.

The item response theory parameter estimates from the two-parameter generalised partial credit model are presented in Table 4. Discrimination for all items is acceptable, ranging from moderate to very high [18] with all discrimination parameters significant at *p* < 0.001. Threshold estimates are reported in units of theta, with 0 representing the population mean and each unit representing a one SD change. Most threshold estimates were negative, which may be interpreted that responses of less than “a lot” are indicative of levels of family functioning that are lower than the population mean (or for items 3, 5 and 6, responses of less than “a fair bit”).

**Table 4:** Item response theory parameter estimates from the two-parameter generalised partial credit model

Item	Discrimination (95% CI)	Threshold 1: 2 vs 1 (95% CI)	Threshold 2: 3 vs 2 (95% CI)	Threshold 3: 4 vs 3 (95% CI)
We get on together and cope in the hard times	2.32 (2.16, 2.49)	−1.67 (− 1.76, − 1.58)	− 0.84 (− 0.90, − 0.78)	− 0.23 (− 0.28, − 0.18)
We celebrate special days/events	2.29 (2.13, 2.44)	− 1.57 (− 1.66, − 1.49)	− 0.74 (− 0.80, − 0.68)	−0.20 (− 0.25, − 0.15)
We talk with each other about the things that matter	4.08 (3.77, 4.39)	−1.51 (− 1.57, − 1.44)	−0.67 (− 0.72, − 0.63)	0.01 (− 0.03, 0.05)
We are always there for each other	4.71 (4.31, 5.11)	−1.64 (− 1.71, − 1.57)	−0.89 (− 0.94, − 0.85)	−0.30 (− 0.34, − 0.26)
We manage money well	1.10 (1.03, 1.18)	−1.71 (− 1.83, − 1.58)	−0.66 (− 0.75, − 0.58)	0.46 (0.38, 0.54)
We have common interests	2.36 (2.21, 2.52)	−1.58 (− 1.66, − 1.50)	−0.67 (− 0.72, − 0.61)	0.28 (0.23, 0.32)
People are accepted for who they are	2.18 (2.03, 2.33)	−1.87 (− 1.98, − 1.76)	−1.10 (− 1.17, − 1.04)	− 0.35 (− 0.40, − 0.29)
We have good support from mob	0.79 (0.73, 0.85)	−0.65 (− 0.78, − 0.52)	−0.37 (− 0.50, − 0.24)	−0.31 (− 0.43, − 0.18)
We have family knowledge and traditions that we pass on to our children	0.62 (0.57, 0.67)	−1.10 (− 1.25, − 0.94)	0.53 (0.37, 0.68)	−0.38 (− 0.54, − 0.22)

The threshold estimates indicate that all items except two have monotonic increases in the quality of family functioning as responses increase from “not at all” to “a lot”. The item “We have good support from mob” has overlaps in confidence intervals for all three thresholds, indicating that although higher responses tended to be associated with greater family functioning, responses were not well differentiated. Furthermore, the item “We have family knowledge and traditions that we pass on to our children” has inconsistent thresholds, with the second threshold greater than the third, and overlap between thresholds. Again, this suggests a lack of differentiation across the response scale, which may reflect inconsistency in responding patterns among groups of respondents (Table 4).

#### Convergent validity

The FFM demonstrates evidence of convergent validity through strong associations with good family financial security and experience of pain.

Good family financial security is reported by 43.0% of participants. The prevalence of good family financial security is significantly higher among those with moderate, high and very high levels of family functioning compared to those with low family functioning (PR = 1.34, 95%CI = 1.24-1.44 for moderate family functioning; PR = 1.58, 95%CI = 1.47-1.70 for family functioning; PR = 1.61, 95%CI = 1.50-1.73 for very high family functioning) (Table 5; Supplementary file 2, Fig. S1).

**Table 5:** Association between the FFM and good family financial security and pain (convergent validity assessment)

Level of family functioning	Without outcome n(%)	With outcome n(%)	PR of outcome (95%CI)
**Outcome: Good family financial security**			
**FFM (score)**			
Low (9 - ≤24)	1385 (65.7)	724 (34.3)	1 (base)
Moderate (> 24 - ≤29)	1120 (54.1)	952 (46.0)	1.34 (1.24, 1.44)
High (> 29 - ≤33)	917 (45.7)	1091 (54.3)	1.58 (1.47, 1.70)
Very high (> 33 - 36)	786 (44.7)	972 (55.3)	1.61 (1.50, 1.73)
**Outcome: Pain**			
**FFM (score)**			
Low (9 - ≤24)	569 (25.5)	1666 (74.5)	1 (base)
Moderate (> 24 - ≤29)	693 (32.0)	1471 (68.0)	0.91 (0.88, 0.95)
High (> 29 - ≤33)	802 (38.6)	1278 (61.4)	0.82 (0.79, 0.86)
Very high (> 33 - 36)	801 (43.0)	1063 (57.0)	0.77 (0.73, 0.80)

No pain is reported by 32.9% of participants. The prevalence of experiencing any level of pain is significantly lower among those with moderate, high and very high levels of family functioning compared to those with low family functioning (PR = 0.91, 95%CI = 0.88-0.95 for moderate family functioning; PR = 0.82, 95%CI-0.79-0.86 for high family functioning; PR = 0.77, 95%CI = 0.73-0.80 for very high family functioning) (Table 5; Supplementary file 2, fig. S2). A dose-response relationship is apparent, where increases in family functioning are associated with decreases in pain.

#### Divergent validity

The FFM demonstrates evidence of divergent validity, with no association with diagnosis of CVD. 10.8% of participants report a lifetime diagnosis of CVD. The prevalence of self-reported heart disease is not significantly different among participants with moderate (PR = 0.96; 95%CI = 0.81-1.14), high (PR = 0.92; 95%CI = 0.78-1.09) or very high family functioning (PR = 1.06; 95%CI = 0.89-1.25) compared to low levels of family functioning (Table 6; Supplementary file 2, fig. S3).

**Table 6:** Association between the FFM and CVD diagnosis (divergent validity assessment)

Level of family functioning	No CVD diagnosis n(%)	CVD diagnosis n(%)	PR of outcome (95%CI)
**FFM (score)**			
Low (9 - ≤24)	2092 (89.0)	259 (11.0)	1 (ref)
Moderate (> 24 - ≤29)	2008 (89.4)	238 (10.6)	0.96 (0.81, 1.14)
High (> 29 - ≤33)	1929 (89.9)	218 (10.2)	0.92 (0.78, 1.09)
Very high (> 33 - 36)	1733 (88.4)	228 (11.6)	1.06 (0.89, 1.25)

## Discussion

Family functioning is important to Aboriginal and Torres Strait Islander peoples as family and kinship networks are a key aspect of culture [1]. The Mayi Kuwayu Study FFM, adapted from the WAACHS family functioning scale, intends to measure family functioning at the national Aboriginal and Torres Strait Islander population. Its nine items were initially created from a literature review on family and resilience protective factors, with wording developed with Aboriginal health professionals [7]. The scale was subsequently tested through a comprehensive face validity assessment in 28 Aboriginal and Torres Strait Islander focus groups during the Mayi Kuwayu Study questionnaire development [9, 10]. No additional family functioning concepts were identified by focus group participants. This may indicate that the family and resilience protective factors that the WAACHS was created from sufficiently capture concepts of family functioning important to Aboriginal and Torres Strait Islander peoples. Although the WAACHS was developed for a specific sub-population (Western Australian families with Aboriginal children), our study demonstrates that a modified version has applicability for the general Aboriginal and Torres Strait Islander population at the national level.

This is the first time that a comprehensive psychometric assessment has occurred for a family functioning measure to be used in the Aboriginal and Torres Strait Islander adult population. With emerging evidence demonstrating that family-centred approaches in healthcare improve health and wellbeing [5], the FFM can be used for the valid monitoring of family functioning over time at the Aboriginal and Torres Strait Islander population level.

Our results demonstrate clear evidence that the scale is a unidimensional measure of family functioning, is an acceptable measure within the target population, with strong internal validity (Cronbach’s alpha > 0.90). The FFM showed strong evidence of convergent validity, with strong associations of family functioning found with established drivers of family functioning: good family financial security and experience of pain. We also found evidence of divergent validity, with no association between family functioning and CVD.

Construct validity assessment through CFA had mixed results for the fit of the model. Modification indices indicated model improvement if two pairs of error terms were correlated (“support from mob” and “knowledge”). The model was rerun with the error terms correlated. The fit of the model was improved, with SRMR = 0.026, CFI = 0.976, TLI = 0.966 all within guidelines for good fit, while RMSEA = 0.073 indicated adequate fit. Overall, there was good indication of uni-dimensionality, although evidence that some items provide redundancy in the scale. The potential issues with two items (“support from mob” and “knowledge”) were based on higher non-response rates, local dependence (which suggests redundancy), and a lack of differentiation between responses on the latent construct. This could suggest that the items are ambiguous, difficult to understand, or are interpreted differently by certain groups within the sample. However, our decision on these two items is not a purely data-driven decision; we also consider expert and end-user consensus.

Consultation with experts in family wellbeing and end-users, including Aboriginal and Torres Strait Islander researchers and the Thiitu Tharrmay Aboriginal and Torres Strait Islander Reference Group, agreed that removing these items from the scale would lead to a loss of coverage for key topic areas relevant to family functioning. Members of Thiitu Tharrmay stated that removal of these items would not give a clear and true picture of family functioning for Aboriginal and Torres Strait Islander peoples. Thus, despite some concerns, given the sound psychometric properties of the measure and the importance of assessing the relationship of support from mob and family traditions, we recommend that the full scale be retained. Members of Thiitu Tharrmay also recommend further research in creating certainty around these two items. Our team will undertake further research in this area. This will include cognitive interviewing to explore the processes by which people respond to these items, and whether the scale would be more robust if these items were omitted or revised, or had clarifying statements as present in the WAACHS, to improve understanding and construct relevance. Further research may also consider whether there are meaningful thresholds of family functioning that can be derived from the scale, and whether these align with the WAACHS thresholds.

### Limitations

The Mayi Kuwayu Study is a national longitudinal study which was designed to capture a diversity of experiences, not to be representative of the total Aboriginal and Torres Strait Islander population. However, within-sample comparisons are generalisable to the whole population, and representativeness of the population is not necessary for reliable estimates. To produce generalisable results, variability in variables studied and their confounders is necessary [20]. As the Mayi Kuwayu Study meets these acceptable criteria, the non-representativeness of the sample will not impact on the robustness of reliability and validity testing.

One potential limitation of this study is the inability to directly compare the FFM to the WAACHS family functioning scale from which it was developed to ascertain if it has better psychometric properties. We recommend this as a future research direction. We also recommend further research examining questions such as how family functioning relates to individual wellbeing, whether the FFM is sensitive to change over time (longitudinally and in response to intervention), and how family functioning impacts on subsequent health outcomes. Another potential limitation is the high number of “unsure” responses, which may be problematic for loss of data particularly in smaller samples. Imputation of the mean score for participants with one unsure or missing response reduces this high number and allows these participants to be included in the study. As noted, cognitive interviewing to interpret the meaning of “unsure” responses and finding solutions to appropriately coding these data would also be beneficial.

Finally, there are limitations in our use of simple mean imputation, as multiple imputation is considered a most robust approach to dealing with missing data. When selecting the of method for dealing with missing data, researchers should “balance validity, ease of interpretability for readers, and analysis expertise of the research team” [11]. While multiple imputation is a more robust and accurate method of dealing with missing data than simple mean imputation, given our goal was to enable widespread use of the FFM in the Aboriginal and Torres Strait Islander population, individual mean imputation is appropriate as it will enable widespread use of the measure while still maintaining statistical rigour [11].

## Conclusion

This work is important, as measures that are used in research must be validated for the population they are to be used within, to ensure they are meaningful to the population and measure what they intend to measure. Our findings provide evidence that the Mayi Kuwayu Study FFM is a valid measure of family functioning in the Aboriginal and Torres Strait Islander population, opening up avenues for research and monitoring with an appropriate and valid measurement of Aboriginal and Torres Strait Islander family functioning, where data can be used to advocate for programs and services. The FFM is available for use by communities, researchers and policy makers with appropriate reference (Supplementary file 3).

## Supplementary Information

**Additional file 1.**

**Additional file 2.**

**Additional file 3.**

## Data Availability

The data that support the findings of this study are available through application to the Mayi Kuwayu Data Governance Committee (MKDGC). Restrictions apply to the availability and use of these data, which were used under license for the current study, and so are not publicly available. The MKDGC oversees and approves applications for data use, in order to maintain the confidentiality of participants, and ensure that all studies using Mayi Kuwayu Study data are protective of Aboriginal and Torres Strait Islander data, cultures, and sovereignty. Data are available with permission of the MKDGC. The application process is detailed here: mkstudy.com.au/overview/ The corresponding author can be contacted for assistance with data requests.
