# Supplementary material for: The development and validation of a family functioning measure for Aboriginal and Torres Strait Islander adults
Source: BMC Public Health. 2022 Oct 28;22:1976. doi: 10.1186/s12889-022-14363-7 (PMC9615397; doi:10.1186/s12889-022-14363-7)
Supplement: Supplementary file 1 — Additional file 1. [file 12889_2022_14363_MOESM1_ESM.docx]

**Supplementary file 1**

Table S1: Summary of Mayi Kuwayu Study focus groups

| **Focus Group** | **State/Territory** | **Remoteness** | **Type of Organisation** | **Number of Participants** |
| --- | --- | --- | --- | --- |
| 1 | NSW | Regional |  | 25 |
| 2 |  |  |  |  |
| 3 |  |  |  |  |
| 4 |  |  |  |  |
| 5 | NSW | Regional |  | 9 |
| 6 | NT | Remote |  | 7 |
| 7 | NSW | Regional |  | 9 |
| 8 | ACT | N/A | National Conference | 25 |
| 9 |  |  |  |  |
| 10 |  |  |  |  |
| 11 | NSW | Regional |  | 7 |
| 12 | WA | Regional |  | 8 |
| 13 |  |  |  | 13 |
| 14 | QLD | Regional |  | 4 |
| 15 | QLD | Remote |  | 5 |
| 16 | NT | Regional |  | 4 |
| 17 | SA | Remote |  | 7 |
| 18 | ACT | Urban |  | 5 |
| 19 | WA | Regional |  | 10 |
| 20 | WA | N/A | National Conference | 6 |
| 21 |  |  |  | 9 |
| 22 |  |  |  | 5 |
| 23 |  |  |  | 7 |
| 24 |  |  |  | 8 |
| 25 |  |  |  | 4 |
| 26 |  |  |  | 9 |
| 27 | TAS | Urban |  | 8 |
| 28 | NT | Remote |  | 3 |
| Total Number of Participants | | | | 197 |

Table S2: Detailed variable definitions

| **Demographics** | |
| --- | --- |
| Age group | Age in years is calculated based on “date of entry” (i.e. date of completion of the survey) minus self-reported “date of birth”, rounded to one decimal place. Implausible values are recoded to “.” (missing).  Age is recoded and analysed as a categorical variable in 10-year groups starting from age 16 (minimum age), up to 65 years: 16-24, 25-34, 35-44, 45-54, 55-64, ≥65. |
| Gender | Participants are asked “What is your gender” with response options (1) male, (2) female, (3) other. Missing values are coded as “.”  There are a small number of persons identifying as other genders, so the binary variable is used for analyses: (1) male (2) female, (.) missing/other. |
| Remoteness | Remoteness is based on self-reported postcode of home address, and is analysed as a categorical variable with 3 categories from the ABS Remoteness categories: (1) major city (2) inner and outer regional (3) remote and very remote. Missing values are coded as “.” |
| Indigeneity | Participants are asked if they are (1) Aboriginal (2) Torres Strait Islander (3) Both Aboriginal and Torres Strait Islander (4) Neither Aboriginal nor Torres Strait Islander. We remove participants who are neither Aboriginal nor Torres Strait Islander. |
| **Convergent Validity** | |
| Family financial security | Participants are asked: “Which words best describe your family’s money situation?” with response options coded as:  (1) We have a lot of savings  (2) We have some savings  (3) We have just enough money to get us to the next payday  (4) We run out of money before payday  (5) We are spending more than we get  (6) Unsure  A binary variable is created: 0 “No savings” (response options 3,4,5) and 1 “Has savings” (response options 1,2). “Unsure” is recoded to missing. |
| Pain level | Question: “In the last 4 weeks about how often did you feel pain?” with response options coded as:  (1) All of the time  (2) Most of the time  (3) Some of the time  (4) A little of the time  (5) None of the time  Binary variable created: 0 “No pain” (response option 5) and 1 “Some pain” (response options 1,2,3,4).  Validation work on this pain question (Milnes, in progress) demonstrates that pain reported is not only physical pain, but also encompasses holistic aspects of social and emotional wellbeing. Domains of pain reported in this question include: body, mind/emotions, trauma, family and kinship, community, culture, Country, financial, aging and spirit/spirituality. |
| **Divergent Validity** | |
| CVD | Question: “Has a doctor ever told you that you have heart disease?” with response options coded as:  (0) Not selected  (1) Selected  Binary variable created: 0 “No CVD diagnosis” (response option 0) and 1 “CVD diagnosis” (response option 1). |

Table S3: Distribution of participants by demographic characteristics and assessment of family functioning scores with imputation compared to family wellbeing scores without imputation

|  | **With Imputation** | | | **Without Imputation** | | |
| --- | --- | --- | --- | --- | --- | --- |
|  | n | % | Mean score (95%CI) | n | % | Mean score (95%CI) |
| **Total family functioning score** |  |  | 27.64 (27.48, 27.79) |  |  | 27.68 (27.55, 27.82) |
| **Age group (years)** |  |  |  |  |  |  |
| 16-24 | 755 | 10.2 | 27.33 (26.83, 27.82) | 881 | 10.1 | 27.48 (27.03, 27.93) |
| 25-34 | 1,050 | 14.1 | 27.87 (27.47, 28.26) | 1,160 | 13.3 | 27.85 (27.48, 28.23) |
| 35-44 | 1,152 | 15.5 | 27.68 (27.31, 28.06) | 1,283 | 14.7 | 27.74 (27.39, 28.09) |
| 45-54 | 1,420 | 19.1 | 27.29 (26.93, 27.64) | 1,649 | 18.9 | 27.19 (26.86, 27.52) |
| 55-64 | 1,658 | 22.3 | 27.36 (27.03, 27.68) | 2,000 | 23.0 | 27.50 (27.21, 27.79) |
| ≥65 | 1,190 | 16.0 | 28.40 (28.02, 28.78) | 1,481 | 17.0 | 28.45 (28.12, 28.79) |
| Missing | 215 | 2.9 | - | 251 | 2.9 | - |
| **Gender** |  |  |  |  |  |  |
| Male | 2,759 | 37.1 | 27.32 (27.07, 27.57) | 3,274 | 37.6 | 27.34 (27.11, 27.57) |
| Female | 4,517 | 60.7 | 27.85 (27.65, 28.05) | 5,243 | 60.2 | 27.92 (27.74, 28.10) |
| Other genders | 6 | 0.1 | 22.05 (18.55, 26.45) | 7 | 0.1 | 23.46 (19.63, 27.30) |
| Missing | 158 | 2.1 | - | 181 | 2.1 | - |
| **Level of remoteness** |  |  |  |  |  |  |
| Major city | 3,019 | 40.6 | 27.10 (26.87, 27.34) | 3,594 | 41.3 | 27.23 (27.01, 27.45) |
| Regional | 3,523 | 47.4 | 27.61 (27.39, 27.83) | 4,119 | 47.3 | 27.66 (27.45, 27.86) |
| Remote and very remote | 874 | 11.8 | 29.60 (29.18, 30.01) | 959 | 11.0 | 29.55 (29.15, 29.94) |
| Missing | 24 | 0.3 | - | 33 | 0.4 | - |
| **Indigeneity** |  |  |  |  |  |  |
| Aboriginal | 6,784 | 91.2 | 27.59 (27.43, 27.75) | 7,946 | 91.3 | 27.64 (27.49, 27.78) |
| Torres Strait Islander | 239 | 3.2 | 28.47 (27.58, 29.36) | 270 | 3.1 | 28.58 (27.75, 29.40) |
| Aboriginal and Torres Strait Islander | 310 | 4.2 | 27.92 (27.16, 28.67) | 355 | 4.1 | 27.99 (27.29, 28.69) |
| Missing | 107 | 1.4 | - | 134 | 1.5 | - |

**Supplementary file 4**

Table S4: Proportion of “unsure” responses to family wellbeing items across demographic information

|  | **Cope*^** | | **Celebrate*^** | | **Talk*^** | | **There*^** | | **Money*^** | | **Interest*^~** | | **Accepted*^** | | **Mob^~** | | **Pass^~** | |
| --- | --- | --- | --- | --- | --- | --- | --- | --- | --- | --- | --- | --- | --- | --- | --- | --- | --- | --- |
|  | Unsure | Sure | Unsure | Sure | Unsure | Sure | Unsure | Sure | Unsure | Sure | Unsure | Sure | Unsure | Sure | Unsure | Sure | Unsure | Sure |
|  | **%** | | | | | | | | | | | | | | | | | |
| **Age** |  |  |  |  |  |  |  |  |  |  |  |  |  |  |  |  |  |  |
| 16-24 | 14.9 | 10.6 | 16.3 | 10.6 | 18.1 | 10.6 | 15.1 | 10.6 | 14.2 | 10.5 | 17.2 | 10.4 | 13.1 | 10.6 | 10.5 | 10.7 | 13.9 | 10.4 |
| 25-34 | 10.9 | 13.4 | 14.1 | 13.4 | 12.6 | 13.4 | 10.1 | 13.4 | 11.0 | 13.5 | 8.8 | 13.5 | 10.4 | 13.5 | 9.3 | 14.2 | 10.9 | 13.7 |
| 35-44 | 10.9 | 14.8 | 11.1 | 14.8 | 11.0 | 14.7 | 14.3 | 14.7 | 11.0 | 14.9 | 10.0 | 14.9 | 12.2 | 14.8 | 9.7 | 15.7 | 10.5 | 15.2 |
| 45-54 | 16.7 | 19.3 | 18.5 | 19.2 | 19.7 | 19.2 | 19.3 | 19.2 | 20.6 | 19.2 | 18.4 | 19.3 | 18.0 | 19.2 | 17.5 | 19.6 | 16.3 | 19.5 |
| 55-64 | 26.4 | 23.9 | 17.8 | 24.0 | 21.3 | 23.9 | 21.9 | 23.9 | 23.8 | 23.9 | 24.8 | 23.9 | 27.5 | 23.8 | 29.2 | 23.0 | 26.6 | 23.6 |
| 65+ | 20.1 | 18.1 | 22.2 | 18.1 | 17.3 | 18.2 | 19.3 | 18.2 | 19.5 | 18.1 | 20.8 | 18.0 | 18.9 | 18.2 | 23.9 | 16.9 | 21.9 | 17.7 |
| *Total* | 100.0 | 100.0 | 100.0 | 100.0 | 100.0 | 100.0 | 100.0 | 100.0 | 100.0 | 100.0 | 100.0 | 100.0 | 100.0 | 100.0 | 100.0 | 100.0 | 100.0 | 100.0 |
| **Gender** |  |  |  |  |  |  |  |  |  |  |  |  |  |  |  |  |  |  |
| Men | 54.9 | 38.4 | 53.0 | 38.5 | 53.9 | 38.5 | 54.6 | 38.5 | 46.1 | 38.5 | 51.0 | 38.3 | 46.9 | 38.5 | 39.4 | 38.6 | 40.7 | 38.4 |
| Women | 45.1 | 61.6 | 47.0 | 61.5 | 46.1 | 61.5 | 45.5 | 61.5 | 53.9 | 61.5 | 49.0 | 61.7 | 53.2 | 61.5 | 60.6 | 61.4 | 59.3 | 61.6 |
| *Total* | 100.0 | 100.0 | 100.0 | 100.0 | 100.0 | 100.0 | 100.0 | 100.0 | 100.0 | 100.0 | 100.0 | 100.0 | 100.0 | 100.0 | 100.0 | 100.0 | 100.0 | 100.0 |
| **Remoteness** |  |  |  |  |  |  |  |  |  |  |  |  |  |  |  |  |  |  |
| Major City | 32.6 | 41.6 | 28.8 | 41.7 | 26.5 | 41.6 | 31.2 | 41.6 | 31.5 | 41.8 | 33.6 | 41.8 | 32.6 | 41.7 | 47.6 | 40.5 | 44.8 | 41.2 |
| Regional | 59.7 | 47.5 | 61.9 | 47.5 | 65.9 | 47.6 | 62.4 | 47.6 | 55.8 | 47.5 | 52.9 | 47.6 | 55.5 | 47.6 | 49.8 | 47.4 | 50.6 | 47.5 |
| Remote | 7.7 | 10.9 | 9.4 | 10.8 | 7.6 | 10.8 | 6.4 | 10.8 | 12.7 | 10.7 | 13.5 | 10.6 | 11.9 | 10.7 | 2.6 | 12.1 | 4.6 | 11.3 |
| *Total* | 100.0 | 100.0 | 100.0 | 100.0 | 100.0 | 100.0 | 100.0 | 100.0 | 100.0 | 100.0 | 100.0 | 100.0 | 100.0 | 100.0 | 100.0 | 100.0 | 100.0 | 100.0 |

^*significant on gender^

^^significant on remoteness^

^~significant on age^
