# Supplementary material for: The development and validation of a family functioning measure for Aboriginal and Torres Strait Islander adults
Source: BMC Public Health. 2022 Oct 28;22:1976. doi: 10.1186/s12889-022-14363-7 (PMC9615397; doi:10.1186/s12889-022-14363-7)
Supplement: Supplementary file 2 — Additional file 2. [file 12889_2022_14363_MOESM2_ESM.docx]

**Supplementary file 2**

Figure S1: Coefficient plot – prevalence ratio of good family financial security by level of family functioning

Figure S2: Coefficient plot – prevalence ratio of experience of pain by level of family functioning

Figure S3: Coefficient plot – prevalence ratio of CVD diagnosis by level of family wellbeing
