# Supplementary material for: The development and validation of a family functioning measure for Aboriginal and Torres Strait Islander adults
Source: BMC Public Health. 2022 Oct 28;22:1976. doi: 10.1186/s12889-022-14363-7 (PMC9615397; doi:10.1186/s12889-022-14363-7)
Supplement: Supplementary file 3 — Additional file 3. [file 12889_2022_14363_MOESM3_ESM.docx]

**The Mayi Kuwayu Family Functioning Measure**

These questions ask about your family. For each question, mark the circle that best describes how you feel.

| **In my family…** | **Not at all** | **A little bit** | **A fair bit** | **A lot** |
| --- | --- | --- | --- | --- |
| We get on together and cope in the hard times | O | O | O | O |
| We celebrate special days/events | O | O | O | O |
| We talk with each other about the things that matter | O | O | O | O |
| We are always there for each other | O | O | O | O |
| We manage money well | O | O | O | O |
| We have common interests | O | O | O | O |
| People are accepted for who they are | O | O | O | O |
| We have good support from mob | O | O | O | O |
| We have family knowledge and traditions that we pass on to our children | O | O | O | O |

**Family Functioning Measure Scoring**

Response options are scored as: “not at all” (score=1), “a little bit” (score=2), “a fair bit” (score=3), “a lot” (score=4). If a participant is missing on one item only, the mean of their other 8 items replaces the missing item.

A total family functioning score is created by summing responses to the nine items. The total family functioning score is recoded to missing if two or more individual items are “missing”.

Family functioning quartiles are created based on the total family functioning score:

- Low family functioning (scores: 9 to ≤24)
- Moderate family functioning (scores: >24 to ≤29)
- High family functioning (scores: >29 to ≤33)
- Very high family functioning (scores: >33 to 36)
